# Do patients trust the tech? Exploring perception, confidence, and knowledge of innovations in shoulder arthroplasty

**DOI:** 10.1016/j.jseint.2025.101415

**Published:** 2025-12-02

**Authors:** Aghdas Movassaghi, Elizabeth W. Chan, Justin T. Childers, Benjamin T. Lack, Garrett R. Jackson, Clyde Fomunung, Roya Osswald, Vani J. Sabesan

**Affiliations:** a Michigan State University College of Human Medicine, East Lansing, MI, USA; b Campbell University Jerry M. Wallace School of Osteopathic Medicine, Buies Creek, NC, USA; c Charles E. Schmidt College of Medicine, Florida Atlantic University, Boca Raton, FL, USA; d Department of Orthopaedic Surgery, University of Missouri, Columbia, MO, USA; e Houston Methodist Hospital, Houston, TX, USA; f University of Florida, Gainesville, FL, USA; g Orthopedic Center of Palm Beach County, Atlantis, FL, USA

**Keywords:** Shoulder arthroplasty, Preoperative planning, Technology, Computer navigation, Augmented reality, Patient education

## Abstract

**Background:**

As surgical technologies, such as three-dimensional preoperative planning, computer navigation, and augmented reality, become increasingly utilized in shoulder arthroplasty, questions remain about their value from the patient's perspective. While education and patient interest have driven demand and technology adoption in hip and knee arthroplasty, their role in shoulder procedures remains unclear. This study aimed to evaluate patient perceptions of innovative technologies in shoulder arthroplasty and assess whether preoperative education influences confidence, satisfaction, and expectations.

**Methods:**

In this prospective observational study, 87 patients scheduled to undergo shoulder arthroplasty at a single institution completed a preoperative survey assessing demographics, baseline familiarity with surgical technologies, and perceptions of surgeon use of innovative tools prior to seeing their provider. Following this, patients then viewed a standardized educational video on the role of technology in shoulder arthroplasty. Postvideo responses measured changes in confidence, satisfaction, and outcome expectations. Statistical analysis included paired t-tests and analysis of variance to evaluate prepost changes and demographic associations.

**Results:**

Over half of patients (56.3%) were unfamiliar with innovative technologies at baseline, yet 60.9% reported increased confidence in surgeons using it. Most patients (66.7%) preferred the use of advanced planning technologies, though only 41.5% would choose a low-volume surgeon using these tools over a high-volume surgeon using conventional techniques. Improvements in confidence were significantly correlated with higher education and income levels (r = 0.31, *P* = .003). After viewing an educational video, patient confidence in their surgeon increased (*P* = .03), and expectations for improved outcomes (*P* < .001), fewer complications (*P* < .001), less pain (*P* < .001), and faster recovery (*P* < .001) significantly rose. Despite favorable perceptions, 62.1% of patients were unwilling to pay more, travel further, or wait longer to receive care involving innovative technologies.

**Conclusion:**

Targeted preoperative education on surgical technology may improve patient confidence, strengthen perceptions of surgeon competency, and elevate expectations of care. While enthusiasm for advanced tools was observed following education, broader adoption may still be influenced by cost and accessibility. These findings support the role of brief, technology-focused education in enhancing the overall patient experience in shoulder arthroplasty.

Shoulder arthroplasty can present significant technical challenges, particularly in cases involving glenoid deformity, limited exposure, or complex implant positioning.^5^^,^^11^^,^^22^^,^^33^ Conventional techniques that rely on two-dimensional imaging and manual instrumentation may fall short in accurately addressing these anatomical complexities. To overcome these challenges, innovative technologies such as three-dimensional (3D) preoperative planning, computer-assisted navigation, and augmented reality (AR) have been developed to enhance surgical precision, improve alignment, and optimize outcomes.^11^^,^^27^^,^^29^^,^^31^

Despite their potential, the widespread adoption of these technologies remains limited. Barriers include higher costs, longer setup and operative times, limited access, and the need for additional surgeon training.^2^^,^^7^^,^^11^^,^^18^ While some studies have shown improved accuracy and efficiency, the broader value proposition remains unclear.^12^^,^^13^ In total hip and knee arthroplasty, patient education has been shown to improve trust, reduce anxiety, and support the adoption of new technologies.^5^^,^^8^^,^^16^^,^^19^ In addition, for patients undergoing total knee arthroplasty (TKA), patient education and interest have played a meaningful role in accelerating adoption and supporting the integration of new technologies such as robotics, helping to justify the increased cost.^6^ However, little is known about how patients perceive similar technologies in shoulder arthroplasty, where research and education efforts have lagged behind.

The purpose of this study was to evaluate patient perceptions of advanced technologies in shoulder arthroplasty, such as 3D planning, navigation, and AR, and to assess whether a standardized educational video influences patient preoperative confidence, satisfaction, and expectations. We hypothesized that patients would view these technologies favorably and that education would further improve their perceptions and confidence in their surgical care.

## Materials and methods

### Study design

This prospective observational study was conducted at a single institution between 2022 and 2024. Adult patients (≥18 years) scheduled to undergo shoulder arthroplasty by a fellowship-trained shoulder surgeon were prospectively enrolled during their preoperative clinic visit. This was a consecutive series of adult patients scheduled for shoulder arthroplasty who met inclusion criteria during the study period. All participants provided written informed consent, and the study was approved by the institutional review board. No incentives were offered for participation.

### Data collection

Participants completed a preintervention survey assessing demographics (age, sex, race, income, and education), health literacy, and baseline familiarity with advanced technologies in shoulder arthroplasty. All preintervention measures, including health literacy, were assessed using a self-reported survey designed specifically for this study. They then viewed a standardized educational video explaining the role of surgical technologies such as 3D planning, navigation, and AR. Following the video, participants completed a postintervention survey assessing changes in understanding, confidence in the surgeon, satisfaction with technology use, and expectations regarding surgical outcomes. The complete survey was not derived from previously validated instruments and can be found in full in Supplementary Appendix S1.

### Statistical analysis

Descriptive statistics were conducted using IBM Statistical Package for the Social Sciences Statistics Software (version 28.0; IBM Corp., Armonk, NY, USA). Pearson's chi-square and Fisher's exact tests were used for bivariate analysis of categorical variables. One-way analysis of variance evaluated associations between demographics and patient perceptions. Paired *t*-tests compared changes in confidence, satisfaction, and perceptions of outcomes before and after the educational video. A *P* value <.05 was considered statistically significant.

## Results

### Demographics

Overall, 87 patients were included in the study, of which 51.7% were female with a mean age of 69 years (range: 42-88 years). The sample was predominantly Caucasian (79.3%), followed by African American (9.2%), Hispanic/Latino (8%), Asian (2.3%), and Other (1.1%). The most reported income brackets were $50,000-$74,999 (29.89%) and $75,000-$99,999 (20.69%), and 56.3% of the cohort had at least a bachelor's degree. (Table I).

**Table I:** Patient demographics.

Cohort demographics	N (%)
Total	87
Age (>65)	61 (70)
Male	42 (48.3)
Race/ethnicity	
White/Caucasian	69 (79.3)
Black/African American	8 (9.2)
Hispanic/Latino	7 (8)
Asian/Asian American	2 (2.3)
Other	1 (1.1)
Highest educational degree	
High school/GED degree or less	38 (43.7)
College degree	42 (48.3)
Graduate/professional degree	7 (8)
Annual income	
<US $50,000	30 (34.5)
US $50,000-US $74,999	26 (28.7)
US $75,000-US $99,999	18 (20.7)
>US $100,000	13 (14.9)
Primary health insurance	
Private or commercial	30 (34.5)
Medicare	49 (56.3)
Medicaid	2 (2.3)
Military or veterans	5 (5.7)
None	1 (1.1)
Health literacy	
Extremely confident	46 (52.9)
Very confident	11 (12.6)
Somewhat confident	22 (25.3)
Not so confident	6 (6.9)
Not at all confident	2 (2.3)

*GED*, General Educational Development.

Demographic characteristics of the study cohort (N = 87) undergoing shoulder arthroplasty. Health literacy was measured by self-reported confidence in completing medical forms.

### Baseline perceptions of technology

When participants were asked about their use of technology, 34.1% indicated they currently use new technologies before other people, and 56.3% were unfamiliar with the use of advanced technologies in shoulder arthroplasty. Of the cohort, 81.4% accurately described preoperative planning as the surgeon using images to create a model of their anatomy to plan the surgery (Table II).

**Table II:** Patient baseline perceptions technology.

Before watching the patient education video	N (%)
General patient perceptions of new technology	
I am skeptical of new technology and use them only when I have to.	15 (17)
I am usually one of the last people I know to use new technology.	25 (28.4)
I usually use new technologies when most people I know do.	30 (34.1)
I like new technologies and use them before most people I know.	12 (13.6)
I love new technologies and am among the first to experiment with and use them.	5 (5.7)
Patient familiarity with the use of innovative technology in orthopedic surgery	
Very familiar	8 (9.2)
Somewhat familiar	30 (34.5)
Not at all	49 (56.3)
Which option do you think most accurately describes innovative technology such as preoperative planning and robotics?	
The surgeon uses images to create a model of your anatomy to plan your surgery	70 (81.4)
The surgeon tells the computer what to do and the computer plans your surgery	14 (16.3)
A robot performs the surgery and a surgeon stands by on the computer	2 (2.3)
The robot and computer perform the surgery and the surgeon is not in the operating room	0

### Impact of technology on preference and confidence in surgeon

Most participants (60.9%) indicated their confidence in a surgeon would increase if innovative technologies were used. In addition, 56.3% of patients believed that surgeons who offer preoperative planning with advanced technology are better than those who do not. However, less than half (41.3%) of patients would prefer a low-volume surgeon who uses innovative technology to a high-volume surgeon using conventional techniques (Table III).

**Table III:** Impact of technology on patient preference and confidence in surgeon.

Question	N (%)
Would your confidence level of your surgeon increase if your surgeon used innovative technology?	
Yes	53 (60.9)
No	34 (39.1)
Do you think surgeons who offer innovative technology are better than those who do not?	
Yes	49 (56.3)
No	38 (43.7)
Would you rather have orthopedic surgery performed by a low-volume surgeon who uses innovative preoperative planning technology or a high-volume surgeon who does not use innovative preoperative planning technology?	
Low-volume surgeon who uses innovative preoperative planning technology	33 (41.3)
High-volume surgeon who does not use innovative preoperative planning technology	47 (58.7)

### Impact of patient educational video on confidence and satisfaction

Before watching the educational video, patients reported high confidence in their surgeons, with an average confidence score of 95.5%, which increased to 97.6% after viewing the video (*P* = .034). Patient satisfaction with knowing their surgeon uses innovative technology was high prior to watching the video (94.6%), with no difference in satisfaction afterward (96.8%) (*P* = .086) (Table IV). Education and income were significantly associated with confidence improvements (*P* = .045 and *P* = .049, respectively), with higher education and income levels positively correlated (*P* = .003).

**Table IV:** Impact of patient education video on confidence and satisfaction.

Question	Before video	After video	*P* value
What is your confidence level in your surgeon?	95.5	97.6	.034∗
How would you rate your satisfaction in knowing that your surgeon uses innovative technology?	94.6	96.8	.086

∗ Indicates statistical significance at *P* < .05.

### Impact of a technology-focused educational video on patient expectations

**Figure 1:**
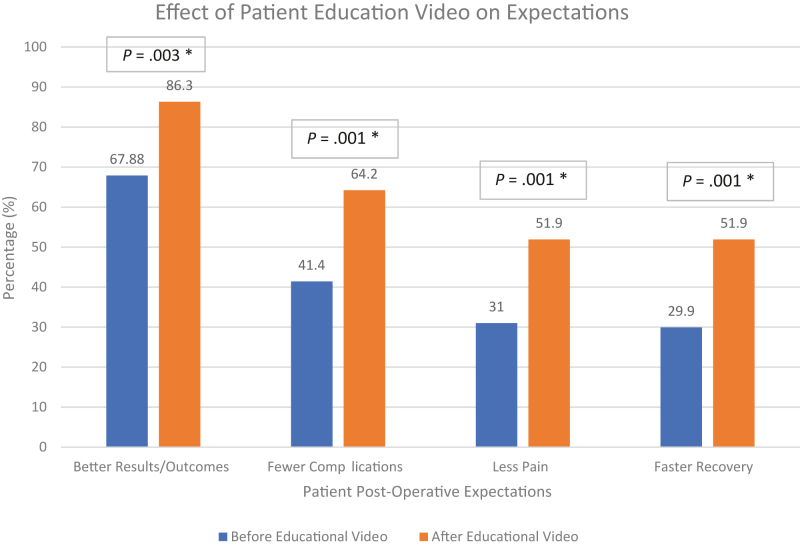
Impact of a patient education video on expectations after shoulder arthroplasty. ∗Indicates statistical significance at *P* < .05.

The education video significantly improved patient expectations for outcomes. Predictions for “better overall results” increased from 67.8% to 86.3% (*P* = .003), “fewer complications” from 41.4% to 64.2% (*P* < .001), “less postoperative pain” from 31.0% to 51.9% (*P* = .001), and “faster recovery” from 29.9% to 51.9% (*P* < .001). The percentage of patients who believed the technology would not have much effect decreased from 14.4% to 8.6% (*P* = .08) (Fig. 1).

### Concerns and barriers to adoptions

**Figure 2:**
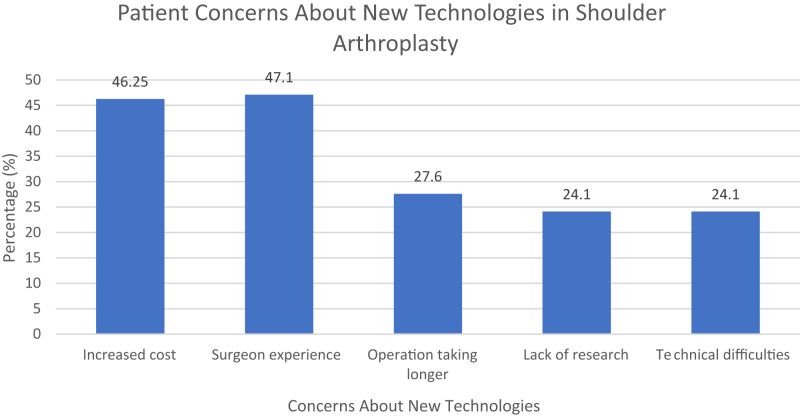
Patient concerns related to the adoption of innovative technologies in shoulder arthroplasty.

Building on these perceptions, patients were also asked about their concerns and willingness to include these technologies in their care. The most common concerns were a lack of surgeon experience (47.1%) and increased cost (46%). Other notable concerns included longer operative times (27.6%) and potential technical difficulties causing complications (24.1%) (Fig. 2). Despite recognizing the valuable potential benefits of innovative technology, most patients (62.1%) were unwilling to pay more, travel further, or wait longer to have innovative technology used in their surgery.

## Discussion

As shoulder arthroplasty continues to incorporate advanced technologies such as 3D planning, navigation, and AR, little is known about how patients perceive these tools or whether education influences their views. While prior studies suggest that patient awareness and education can drive demand for innovation, this has not been well studied in shoulder surgery.^1^^,^^6^^,^^18^ This study introduces data on baseline awareness of innovative technologies, patient preferences and confidence, the effect of a standardized preoperative educational video, and the practical barriers that may limit patient willingness to adopt these advancements.

Despite limited baseline familiarity with innovative technologies, most patients responded favorably to their use, suggesting a general willingness to have the integration of technology in their shoulder surgery. Confidence in the surgeon increased when patients were told these tools would be used, suggesting that even minimal awareness may positively influence trust and demand. However, when asked to choose between a high-volume surgeon and 1 using innovative technologies, patients consistently favored experience over innovation. These findings are consistent with prior studies in total joint arthroplasty. Abdelaal et al^1^ assessed patient perceptions of robotic-assisted TKA and found that most patients had limited understanding of the technology, yet preferred its use. Similarly, Pagani et al^1^^,^^24^ reported that only 51.4% of patients accurately understood the role of robotics, yet both studies concluded that surgeon experience remained the dominant factor in decision-making, even among those who viewed technology positively. This preference for surgeon experience may reflect a desire to maintain control over treatment decisions, with limited understanding of technological advancements reinforcing the perception that such tools reduce, rather than enhance, patient involvement.^30^ This mirrors telemedicine studies, where patients accepted the technology but still preferred in-person orthopedic visits, showing that acceptance does not always translate into preference.^4^^,^^17^^,^^21^ While high surgeon volume has been linked to improved perioperative metrics such as shorter hospital stays and operative times, preoperative planning has been shown to enhance correction of pathology, accuracy of implant placement, and improve outcomes without increasing operative time.^25^^,^^31^^,^^32^ Moreover, evidence suggests that the use of these technologies only extends surgical time during the initial learning curve.^3^^,^^28^

Patient education significantly improved patient confidence and expectations for surgical outcomes, reinforcing its value in supporting informed decision-making.^9^^,^^10^^,^^14^^,^^20^ These findings are consistent with prior work by Fasulo et al,^9^ who demonstrated that preoperative video education improves trust and patient satisfaction for shoulder arthroplasty. While satisfaction scores remained unchanged, this may reflect a ceiling effect, as patients reported high satisfaction levels (94.6%) prior to viewing the educational video. Notably, the impact of education was more pronounced among patients with higher income and education levels, highlighting the influence of health literacy. Over half (52.9%) of the participants reported that they were “extremely confident” in filling out medical forms, which is a sign of health-care literacy.^15^ While this likely contributed to the positive response observed, it also limits generalizability. Standardized educational videos may offer even greater value in more diverse or less health-literate populations; however, future studies need to evaluate this specifically.^26^

Despite favorable perceptions of innovative technologies, patients expressed clear concerns, most commonly related to surgeon experience, increased cost, and potential for longer operative times. While 66.7% valued the use of technology, 62.1% were unwilling to pay more, travel farther, or wait longer to have it included in their surgery. This suggests that while patients appreciate technological advancements, they prioritize minimizing financial and logistical burdens. In addition, these concerns likely reflect the perceived learning curve associated with new surgical technologies, wherein limited surgeon familiarity or training with them may be viewed by patients as increasing procedural risk or uncertainty, thereby serving as a barrier to broader adoption. However, recent studies suggest these technologies may reduce costs and increase efficiency. Sheth et al^31^ demonstrated that preoperative planning significantly decreased intraoperative costs, specifically a reduction in the number of sterilized trays utilized during surgery. Similarly, Novak et al^23^ found that computer-assisted surgery for TKA was cost-effective and, in some cases, cost-saving, though this depended on system costs, alignment accuracy, and revision rates. As these technologies become more integrated, they may offer both clinical and economic value.

These findings suggest that patients are generally receptive to innovative technologies when provided with clear, preoperative education about their role in surgery. Education appears to be a low-cost, scalable intervention that can improve patient confidence and align expectations. Given that higher patient confidence has been associated with improved postoperative outcomes in shoulder arthroplasty, the downstream clinical benefits of education may extend beyond perception alone.^2^ Incorporating standardized patient education on diagnosis, surgical planning, and technology may enhance the patient experience, increase demand, and as a result, support broader adoption of these innovations in shoulder arthroplasty. Future large-scale, multicenter studies correlating patient perceptions with clinical outcomes are necessary and may provide a clearer context to how a patient's understanding of these technologies influences surgical outcomes.

### Limitations

This study is not without its limitations. First, patients lacked a strong understanding of the technologies and responded ambivalently to some survey items. While the educational video improved confidence and outcome expectations, we did not assess comprehension or whether patients felt the video improved their actual knowledge base. Another limitation is that all participants had already decided to proceed with surgery, which may not reflect the perceptions of patients who are still considering surgery. In addition, there is a potential social desirability bias associated with asking patients during their preoperative visit about their confidence in or satisfaction with their surgeon. Our study cohort also demonstrated relatively high health literacy, likely reflective of the region's demographics, which may limit the generalizability of these findings. Broader sampling across diverse populations would strengthen future investigations. Future studies should explore perceptions and educational strategies in more diverse patient populations.

## Conclusion

While most patients are unfamiliar with innovative technologies used in shoulder surgery, this study suggests that patients may prefer its use, gain confidence in surgeons who use it, and anticipate better outcomes after education about these technologies. However, cost and logistical concerns remain key barriers to acceptance. Incorporating targeted preoperative education may improve patient expectations and engagement while supporting the value proposition of advanced surgical tools.

## Disclaimers:

Funding: This study was supported by a research grant received from 10.13039/100008894Stryker.

Conflicts of interest: Vani J. Sabesan, MD, reports consulting relationships with Stryker, DePuy Synthes, and restor3d. In addition, Stryker provided funding for this study. Her immediate family and affiliated research foundations have no additional relevant disclosures. All the other authors, their immediate families, and any research foundation with which they are affiliated have not received any financial payments or other benefits from any commercial entity related to the subject of this article.
