# Supplementary material for: Do patients trust the tech? Exploring perception, confidence, and knowledge of innovations in shoulder arthroplasty
Source: JSES Int. 2025 Dec 2;10(2):101415. doi: 10.1016/j.jseint.2025.101415 (PMC12814059; doi:10.1016/j.jseint.2025.101415)
Supplement: Supplementary Appendix S1 [file mmc1.docx]

1. **Initial Evaluation: Patient Characteristics**^5–8^ (Preop)
2. What is your Study ID? (Enter your first and last name if you do not know it) _________
3. What is today’s date? ___________
4. What is your age (years)? ______
5. What is your gender?

Male

Female

Other (please specify):_________

1. Is English your first language?

Yes

No

1. What region of the United States do you live in? (*Please select one*)

Northeast

South

Midwest

West

1. What is your marital status? (*Please select one*)

Single

Married

Divorced/Separated

Widowed

1. What is your highest educational degree? (*Please select one*)

Did not finish high school

High school/GED degree

College degree (e.g., BS, BA)

Graduate degree (e.g., MBA, MS, MD, JD, PhD, PharmD)

1. What is your annual income? (*Please select one*)

Under $15,000

Between $15,000 - $29,999

Between $30,000 - $49,999

Between $50,000 - $74,999

Between $75,000 - $99,999

Between $100,000 - $150,000

Over $150,000

1. What is your race/ethnicity? (*Please select one*)

White or Caucasian

Black or African American

Hispanic or Latino

Asian or Asian American

American Indian or Alaska Native

Native Hawaiian or other Pacific Islander

Other (please specify): _________

1. How confident are you at filling out medical forms by yourself? (*Please select one*)

Extremely confident

Very confident

Somewhat confident

Not so confident

Not at all confident

1. What is your primary health insurance? (*Please select one*)

Private or commercial

Medicare

Medicaid

Military or veterans

None

1. Do you work in healthcare?

Yes

No

1. How is your overall health status? (*Please select one*)

Poor or fair

Good

Very good

Excellent

1. When it comes to technology, what best describes you? (*Please select one*)

I am skeptical of new technologies, and use them only when I have to

I am usually one of the last people I know to use new technologies

I usually use new technologies when most people I know do

I like new technologies and use them before most people I know

I love new technologies and am among the first to experiment with and use them

1. **Initial Evaluation: Public Perception of Preoperative Planning, Confidence Level, Outcomes**^7^ (**Preop**)
2. What is your confidence level in your surgeon? (Rate from 0-100% with 100% being extremely confident) ______________
3. How would you rate your satisfaction in knowing that your surgeon uses 3D technology for preoperative planning? (Rate from 0% to 100% with 100% being extremely satisfied)_____________
4. Are you familiar with the use of 3D technology for preoperative planning in orthopedic surgery? (*Please select one*)

Very familiar

Somewhat familiar

Not at all

1. How did you hear about the use of 3D technology for preoperative planning in orthopedic surgery? (*Please select all that apply*)

Television

Family/friends

Internet

Doctor

Being in hospital or having a previous surgery

I have not heard of it before

1. If you were to have orthopedic surgery, would you prefer your surgeon to use 3D innovative technology to plan your surgery? (*Please select one*)

Yes

No

No preference

1. Do you think that the use of 3D technology for preoperative planning would lead to any of the following after your surgery? (*Please check all that apply*)

Better results/outcomes

Fewer complications

Less pain

Faster recovery

I don’t think it’ll have much of an effect

1. Would you be willing to do any of the following to have preoperative planning with 3D technology used during your surgery? (*Please check all that apply*)

Pay more

Travel further

Wait longer for your surgery

No, I am not willing to do any of these

1. Would you prefer preoperative planning with 3D technology to be used during your orthopedic surgery if the results were the same as without 3D technology?

Yes

No

1. Do you think surgeons who offer preoperative planning with 3D technology are doing better than those that do not?

Yes

No

1. Would your confidence level of your surgeon increase if your surgeon used preoperative planning with 3D technology?

Yes

No

1. Did your surgeon use preoperative planning with 3D technology?

Yes

No

1. Would your surgeon’s use of 3D technology with preoperative planning impact your future decision to undergo surgery?

Yes

No

1. Would you rather have orthopedic surgery performed by a low-volume surgeon using 3D preoperative planning technology or a high-volume surgeon that doesn't use 3D preoperative planning technology?

Low-volume surgeon using innovative 3D preoperative planning technology

High-volume surgeon that doesn’t use 3D innovative preoperative planning technology

1. Which option do you think most accurately describes 3D preoperative planning technology? (*Please select one*)

The surgeon uses images to create a model of your anatomy to plan your surgery

The surgeon tells the computer what to do and the computer plans your surgery

A robot performs the surgery and a surgeon stands by on the computer

The robot and computer perform the surgery and the surgeon is not in the operating room

1. What would be your major concern regarding preoperative planning with 3D technology? (*Please check all that apply*)

Increased cost

Operation takes longer

Lack of surgeon experience with preoperative planning

Lack of research to support use of preoperative planning

Technical difficulties in the preoperative plan that causes complications

No concerns

1. **Secondary Evaluation: Patient perception of surgeon after observing the video**^7^ (**Preop**)
   - 1. What is your confidence level in your surgeon? (Rate from 0-100% with 100% being extremely confident) ______________
     2. How would you rate your satisfaction in knowing that your surgeon uses preoperative planning with 3D technology? (Rate from 0% to 100% with 100% being extremely satisfied) _____________
     3. Do you think that the use of preoperative planning with 3D technology would lead to any of the following after your surgery? (*Please check all that apply*)

Better results/outcomes

Fewer complications

Less pain

Faster recovery

I don’t think it’ll have much of an effect
